# Some Remarks on the Febrile Stage of Epidemic Cholera; with the Suggestion of a New Theory of the Propagation and Spread of Cholera

**Published:** 1849-05

**Authors:** E. B. Haskins

**Affiliations:** Clarksville, Tenn.


Art. II.—*Some remarks on the Febrile stage of Epidemic Cholera;* with the suggestion of a new theory of the propagation and spread of Cholera. By Dr. E. B. Haskins, of Clarksville, Tenn.; in a letter to Prof. Yandell.

Dear Sir:—In your review of the Report of the standing Sanitary Committee of the Board of Health of the city of New York, on the subject of Asiatic Cholera &c., I find the following remarks:

"The present epidemic appears to differ in one essential feature from that which invaded our country in 1832. In that, our recollection is, there was little to fear from the subsequent symptoms; if the patient survived the onset of the disease, his recovery was pretty certain. In this, the typhoid stage which follows the primary symptoms is full of danger. According to the report of Dr. Whiting, most of the patients at Staten Island passed through the first attack, and died from subsequent congestion or effusion of the brain. This has been the case in repeated instances in this city. It is a question worthy of serious consideration, whether the free use of opiates, astringents, and stimulants has not contributed to this result."

The typhoid or febrile stage, here spoken of as a new feature in the cholera of the United States by reference to Dr. Jos. Brown's article on cholera, in the Cyc. of Prac. Med., where the literature upon this branch of the subject is well summed up, and ably commented upon, together with his own observations on epidemic cholera, as it was manifested at Sunderland, Eng., in 1832; you will perceive was a very striking and important feature of that disease on the continent of Europe, in England, and to some extent in British India, on its previous visitation of those countries. Respecting the febrile stage of Asiatic cholera, Dr. Brown makes the following strong declaration.

"Now we can positively assert, that we have not met with a single case in England, in which fever did not intervene between the choleric or cold stage and restoration to health, and the results of enquiries addressed to individuals the most observing and the most familiar with the disease in this country, has proved that their experience has coincided with our own." Touching the typhoid character of the fever he says: "In the progress of the fever, the tongue becomes black,.and sordes accumulate about the teeth, the eyes become more and more injected, the intellect more and more torpid, though still the patient can be roused to answer questions, and even may make one or two sensible remarks on his condition; but the instant the conversation ceases, the eyes are turned up in the orbit, expressing through the half closed eyelids the red sclerotica, and the patient is in a state of profound stupor: the urinary secretion is now established, and the urine which was at first dark colored and cloudy, is now limpid and pale; the alvine discharges are darker colored than at first; and throughout the disease there is a deficiency of vascular action, and of temperature, which we have not observed to the same extent in typhus or any other fever. However flushed the countenance may appear, and it is often very considerably so, the temperature of the surface is below the healthy standard, and we have not often found the pulse above ninety. *Typhoid* is not an inappropriate designation of the condition we have attempted to describe." Again, "the duration of snch a febrile stage as we have described, is from a week to ten days. Its termination has been in a considerable majority of instances which have fallen under our observation, fatal. The brain has appeared to us to be the organ mainly affected." Of the *post mortem* appearances Dr. B. says: "In the head are found marks of congestion, and even occasionally of extravasation. Such appearances were not of uniform occurrence in the dissections performed in Hindostan, but they were found very constantly in those made by Davy in Ceylon, and Dr. Keir of Moscow, discovered in the Russian disease, the blood vessels of the brain and its membranes, more or less tinged with blood, particularly toward the base, with a fluid effused into its convolutions, and more or less serum in the lateral ventricles, &c. Dr. Brown also makes extensive extracts from the essays and reports of various observers, in confirmation of his views of the febrile nature of the disease; but one of which, however, I will give, and trust you will pardon me for its length. It is from the "Report of Drs. Russell and Barry to C. C. Greville, Esqr." &c.

"After the blue cold period has lasted from twelve to twenty-four, seldom to forty-eight hours or upwards, the pulse and external heat begin gradually to return, head ache is complained of, with noise in the ears, the tongue becomes more loaded, redder at the tip and edges, and also drier. High colored urine is passed with pain, and in small quantities, the pupil is often dilated, soreness is felt on pressure over the liver, stomach and belly; bleeding by the lancet or leeches is required. Ice to the head gives great relief. In short, the patient is now laboring under a continued fever, not to be distinguished from ordinary fever. A profuse critical perspiration may come on from the second to the third day, and leave the sufferer convalescent; but much more frequently the quickness of pulse and heat of skin continue, the tongue becomes brown and parched, the eyes are suffused and drowsy; there is a dull flush with stupor and heaviness about the countenance much resembling typhus, dark sordes collect about the lips and teeth, sometimes the patient is pale, squalid, and low with the pulse and heat below natural, but, with the typhus stupor, delirium supervenes, and death takes place from the fourth to the eighth day, and even later, in the very individual too, whom the most assiduous attention had barely saved in the first or cold stage."

Drs. Russell and Barry in pointing out the difference, according to their observations, between cholera as it appeared in Europe and in India, say 'Secondly, Restoration to health from the cold stage, without passing through the consecutive fever of any kind, was by far more frequent in India than here (St. Petersburgh); nor did the consecutive fever there assume a typhoid type."

If, therefore, your recollection of the epidemic as it appeared here in 1832, be correct, and that the present prevailing epidemic of our country is marked by a febrile stage, *typhoid* in its type, then it would appear that the American epidemic of 1832 was the Indian variety of malignant cholera, and that the epidemic now prevailing is the European variety of that disease. And hence, an inquiry into the causes which existed—whether the treatment—natural character of the two people, moral, mental and physical,—their political and social condition, the influences of the climate, or what combination of these influences produced the variation in character we have considered—and what it is that has produced the change in the two epidemics in this country, the former assuming the Indian, and the latter the European variety —whether change in treatment (as you have suggested) of atmospheric influences—of habits &c.—and further, whether or not the prevailing epidemic has been marked by the same peculiarities in different regions, that characterized its local development in its former career? And if not, what those changes have been, and the circumstances attending them that may stand related as cause and effect. I say, such an inquiry might lead to no unimportant results.

*Propagation and spread of Cholera.*—Whilst on the subject of Asiatic Cholera, I hope I will not tax your time too heavily, in making some suggestions relative to the mode of propagation and diffusion of this disease,—a subject that has elicited much inquiry and discussion, and certainly does merit, from its weighty bearing upon public, as well as private welfare, a large share of professional attention. For it is a very obvious truth, that a correct knowledge of its mode of propagation, if in a limited degree remediable, may lead to far more valuable results, than can the highest improvements in treatment that so formidable a disease is susceptible of.

Without, therefore, attempting a review of those arguments which have been urged for and against the agency of contagion in the spread of cholera, or stopping to examine other theories that have been advanced by different writers, I will at once invite your attention to a law of organic chemistry, as the basis of a theory, that will come nearer, perhaps, harmonizing all the facts in the history of the origin and progress of this disease, than any that has yet been presented to the professional mind.

It is well known that certain organic elements in a state undergoing decomposition or transformation, when placed in contact, under favorable circumstances, with other organic elements, alike or unlike in chemical constitution, excites in them a metamorphic action, the product of which is either identical in composition with the ferment, or a new compound is formed, differing in composition from the ferment.

Thus, yeast (gluten undergoing decomposition) excites in a solution of vegetable gluten, fermentation, and yeast is reproduced; putrifying animal substances in contact with fresh animal substances, excites putrefactive fermentation; and wood in a state of eremacausis excites the same decomposing action in sound wood placed in contact with it—products identical in chemical constitution with the exciter. Again, yeast in contact with a solution of sugar, excites the vinous fermentation; decomposing casein and other nitrogenous animal substances, excites the lactic fermentation in sugar of milk—products differing in chemical constitution from the exciter.

Liebig explains, in a general way, the laws regulating the identity or non identity of the product of fermentation with the ferment, as follows:

"That a body in the act of decomposition (it may be named the *exciter*,) added to a mixed fluid in which its constituents are contained, can reproduce itself in that fluid, exactly in the same manner as new yeast is produced when yeast is added to liquids containing gluten. This must be more certainly effected when the liquid acted upon contains the body, by the metamorphosis of which the *exciter* has been originally formed;" (Liebig's Organic Chem. in its application to Veg. Phys. and Agriculture. Edited by Playfair, Amer. edition;) and when those constituents are not contained in the fluid a different product from the *exciter* must be the result, of course. Now all that remains is to apply this principle of fermentation, or the laws of *catalysis* (of Berzelius) to organic effluvia, and we at once deduce a theory of the propagation and spread of cholera, from city to city, and country to country in the line of human intercourse, accounting for all the phenomena in its march and development; and clear too of all the objections to the doctrine of contagion, or, the aerial conveyance of an exotic poison.

In order to illustrate our meaning in a few words, we will suppose that in the malarious district of the Delta or the Ganges, where cholera, perhaps, always takes its start, some combination of causes, existing nowhere else —(the nature of which it would be too hypothetical to attempt to define) excites a peculiar fermentation of certain elements of the miasm, spread over that region, the product of which is the cholera poison. From this region, we suppose a portion, enough to act as a ferment, or *exciter* as Liebig terms it, may be conveyed by ships, steamboats and other vehicles of commerce on land and water, and by armies, caravans, and individuals, to other districts and towns infested with organic effluvia, and by the law of catalysis or fermentation reproduce cholera poison. And thus, from city to city, and country to country, may it progress in its career of human destruction, so long as a portion *to act as an exciter* is conveyed in the midst of an effluvium with the *constitution and condition* necessary to its reproduction.

Now this theory is clear of one prominent difficulty that has ever presented itself in the way of the doctrine of contagious propagation.—That in the spread of cholera, it is not to be traced from individual to individual, even where it has owed its origin to the arrival of a vessel, army or caravan; but it usually attacks, simultaneously, a number of individuals in different wards or districts in the same town. It may also be remarked that if it be ascertained to be true, as has been alleged, that influenza, or other milder epidemics, are the forerunners of cholera, this fact will strengthen, rather than weaken the theory here suggested; for in the action of fermentation, the product of the metamorphosis depends upon the chemical constitution of the fluid made the subject of the change; thus, yeast excites in gluten a decomposition that results in the re-production of yeast, whilst it excites in a solution of sugar a fermentation that produces alcohol and carbonic acid. So may the cholera *diastase*, if I may be allowed the figure, when brought in contact with organic effluvia at certain periods, excite a fermentation, resulting in the production of a mild epidemic poison, and at a later period, when other elements, from, perhaps, extraordinary physical causes pervade the effluvium, so as to produce a *certain definite chemical constitution*, the fermentation will result in cholera poison.

If it should be objected that we have assumed too much in supposing that organic effluvia exist as organic compounds, capable of undergoing a process of fermentation, we will appeal to the authority of Liebig for the reasonableness of such an assumption. That philosopher has offered a theory explaining the action of miasms and contagious poisons upon the vital animal fluids, based upon the same assumption of facts. His theory is that miasms and contagious matter excite in the blood a fermentative action resulting in morbid deleterious products. That in the case of miasmata the products are unlike the exciter, and hence no *miasm* is reproduced in the blood—that in the case of contagious matter the product of the fermentation is identical with the exciter and contagious matter is reproduced. (See Liebig's Phys. and Ag. Chem. by Playfair.)

Now as it is requisite for the exciter to be in a state of decomposition or fermentation, before it can produce it in another body, Liebig has evidently assumed that miasms are susceptible of, and do undergo fermentation. And all that astonishes me is that the fertile mind of the Giessen philosopher, that seems often to go in advance of, and anticipate the result of his own well conducted experiments, has not applied so simple a principle of philosophy to the *generation* of epidemic poisons, as well as their *action* upon the *animal fluids;* for if his theory be true, that miasmata do undergo a fermentation capable of exciting that action in the animal fluids, I do not see how we can escape the conclusion that one effluvium in such a state, will disturb the feeble balance of chemical affinities in another, or the same effluvium, resulting in a similar fermentation.

In conclusion we would remark, that the suggestions here preseuted are not offered as at all conclusive; but simply to draw attention to a law of chemical action, that may go far in elucidating, not only the spread of cholera, but also the origin and propagation of such diseases as are termed *common* contagious, as typhus fever, plague,

&c.; and may at length settle the doctrine, so seemingly compatible with philosophic simplicity, that there are no true contagions but those termed *specific*. Besides this view of the subject, if made at all *probable*, will guard city authorities against the heedless neglect of quarantine regulations, simply because the spread of a disease is not explained upon the principles of contagion.

Clarksville, Tenn., March 10th, 1849.

